# Does Quit Smoking Desire Influence E-Cigarette Smoking Behaviour? Malaysian Perspectives

**DOI:** 10.21315/mjms2023.30.3.18

**Published:** 2023-06-27

**Authors:** Sinnappan Santhidran, Thinavan Periyayya, Khairul Anwar Mastor, Idayu Badilla Idris

**Affiliations:** 1 Department of Mass Communication, Universiti Tunku Abdul Rahman, Selangor, Malaysia; 2 Pusat Pengajian Citra Universiti, Universiti Kebangsaan Malaysia, Selangor, Malaysia; 3 Department of Community Health, Faculty of Medicine, Universiti Kebangsaan Malaysia, Selangor, Malaysia

**Keywords:** e-cigarette, desire to quit, perceived health benefit, social impact, social acceptance

## Abstract

Recent years have witnessed an emerging trend of e-cigarette smoking in Southeast Asia. On the basis of Malaysian perspectives, this cross-sectional study explored the relationship between e-cigarette smoking behaviour and variables such as perceived health benefit, the desire to quit, social acceptance, social impact and product usefulness. Individuals aged 17 years old and older were recruited via purposive convenience sampling, yielding a total sample of 503 respondents. Collected data were analysed via partial least squares-structural equational modelling. The results showed that perceived heath benefit (*β* = 0.19, *P <* 0.01), social acceptance (*β* = 0.23, *P <* 0.01) and social impact (*β* = 0.49, *P <* 0.01) positively influence e-cigarette smoking behaviour. No such effect is exerted by the desire to quit smoking (*β* = 0.08, *P <* 0.05) and product usefulness *t* (*β* = −0. 10, *P <* 0.05). Future studies should examine whether demographic variables affect e-cigarette smoking behaviour.

## Introduction

Electronic cigarettes (e-cigarettes) are also known as e-cigs, e-hookahs, mods, vape pens, vapes, tank systems and electronic nicotine delivery systems. In 2011, the number of e-cigarette smokers worldwide amounted to seven million—a figure that escalated more than fivefold in 2018, according to the World Health Organization (WHO). In Southeast Asia, there has been a recently emerging trend of individuals smoking e-cigarettes (1). This considerable marketing opportunity has prompted e-cigarette companies to cater to this region (2).

Adults smoke e-cigarettes for a variety of reasons, among which one of the most important is the desire to quit smoking combustible cigarettes (3, 4). E-cigarette smoking is widely believed to help individuals stop smoking combustible cigarettes despite the absence of conclusive findings (5). Several studies also showed that people who smoke e-cigarettes want to try them out of curiosity, because friends and family members use them or because they are affordable, easily available, convenient to use, healthy and available in good flavours (6). The majority of previous studies on e-cigarette smoking behaviour involved North American and European respondents (4, 7). The problem is that results derived on the basis of Western samples are generalisable to a limited extent to Asian populations because of differences in perspectives and social environments between these regions. To date, few studies have explained the phenomenon of e-cigarette smoking using an inferential statistical approach that focuses on Asian populations. In addition, most e-cigarette studies conducted in Asia were grounded in samples that included school children and adolescents (8–11).

To address these deficiencies, the present research explored the relationship between e-cigarette smoking behaviour and variables such as the desire to quit smoking, perceived health benefit, social acceptance, social impact and product usefulness in Malaysia. The sample comprised individuals aged 17 years old and older.

## Methods

A cross-sectional survey design was employed and the respondents deemed eligible for participation were e-cigarette smokers, individuals aged 17 years old and older, and Malaysian citizens. G*Power analysis showed that a minimum of 138 respondents was the sample size required to achieve a power of 0.95 with a medium effect size (0.15). This research recruited 503 respondents to ensure improved representativeness of the population and accuracy of the findings (12).

### Procedures and Data Collection

An internal review board granted ethical clearance to the researchers. This study was conducted in Selangor, the Federal Territory of Kuala Lumpur and the Federal Territory of Putrajaya, from which participants were recruited via purposive convenience sampling. In each region, five shopping centres where shops or vendors sell e-cigarette-related products were selected for data collection. Prior to such collection, appointed research assistant explained the purpose of the research to shopkeepers before inviting customers to participate in the study. The prospective respondents were then asked to complete an informed consent form to signify their willingness to involve themselves in this work. A paper-and-pencil survey was administered to them, and respondents who completed the survey were given RM20.00 (approximately USD4.65) as compensation for their participation.

Partial least squares-structural equation modelling was carried out to predict variances in e-cigarette smoking behaviours among the participating Malaysian adults. The data were analysed using SmartPLS3 software.

## Results

### Descriptive Statistics

Respondents aged 23 years old–28 years old accounted for the largest group (37%) in the sample. The majority were male (75.5%), and most had diplomas as the highest academic qualification. The greater number of the participants had been smoking e-cigarettes for 6 months to 1 year.

### Measurement Model

The analysis was directed to one dependent variable—e-cigarette smoking behaviour—and five independent variables—perceived health benefit, the desire to quit, social acceptance, social impact and product usefulness. The measurement scale for e-cigarette smoking was adapted from Morean et al. (13) and that for the independent variables was adapted from Soule et al. (14). Both measurement scales are based on a five-point Likert scale ranging from 1 = never to 5 = almost always.

Convergent and discriminant validity were checked with indicator loading, and these generated readings of above 0.7, which is an acceptable level, according to Hair et al. (15) (Table 1). The average variance extracted (AVE) exceeded 0.5 and the composite reliability (CR) was greater than 0.7 (15) (Table 1). The analysis also confirmed that the Cronbach’s alpha was above the threshold suggested by Hair et al. (15) (Table 1). These findings indicate that there were no problems with convergent validity in this work. The heterotrait-monotrait (HTMT) ratio of correlations test was conducted to verify the discriminant validity of the mode. The results showed that it satisfied the threshold of 0.9 and below (16) (Table 2). Therefore, discriminant validity was not a concern in this study.

### Structural Model

Hypothesis significance was tested using bootstrapping methods with 5,000 resamples, significance set at 5% and a one-tailed option. The results showed that out of the five hypotheses, three were supported: H1 (perceived heath benefit; *β* = 0.19, *P <* 0.01), H3 (social acceptance; *β* = 0.23, *P <* 0.01) and H4 (social impact; *β* = 0.49, *P <* 0.01). These positively predicted e-cigarette smoking behaviour. Two hypotheses—H2 (the desire to quit; *β* = 0.08, *P <* 0.05)] and H5 (product usefulness; *β* = −0.10, *P <* 0.05)—were unsupported, suggesting that one cannot predict whether an individual will stop smoking e-cigarettes.

The results indicated that the relationship of perceived health benefit, social acceptance and social impact with e-cigarette smoking behaviour has a small to medium effect size, *f*^2^ = 0.051 to 0.275 (17) (Table 3). The model generated a good prediction, as evidenced by the *Q*^2^ having a value higher than zero (0.369) (Table 3). The findings also showed that three of the variables explained 69.3% of the variance in e-cigarette smoking behaviour; this percentage is sizeable enough to explain the phenomenon of interest (16) (Table 3).

## Discussion

Individual, social and environmental factors, such as social influence, social acceptance and belief in health benefits, strongly influence the smoking behaviours of individuals who use e-cigarettes. Statistical reports showed that the number of e-cigarette users in Malaysia is increasing tremendously every year (18). Countries such as the United Kingdom and New Zealand have capitalised on the positive perceptions of e-cigarettes and their harm reduction benefits to encourage cessation of the more dangerous and harmful tobacco smoking (19). In comparison, with public health and welfare in mind, the Ministry of Health Malaysia has proposed legislation that bans tobacco and e-cigarette smoking for all individuals born after 2005 (20). Although this policy is a favourable step towards a healthy society, many disagree with it because it limits freedom of choice. Some circles also fear that a total smoking prohibition could contribute to the flourishing of the illegal e-cigarette business (20). Therefore, instead of a total ban on e-cigarette smoking, authorities can take a more tactful approach.

### Limitations

The participants in this study were recruited via purposive convenience sampling. Hence, care must be taken when generalising the results. This research can be extended to nationwide samples to enhance generalisation. Demographic variables were not tested in terms of their impact on e-cigarette smoking behaviour. These variables should be included in a future study to monitor their effects on e-cigarette smoking behaviour.

## Conclusion

Given the high level of interest in smoking e-cigarettes among Malaysians and e-cigarette can be used as a potential alternative to discourage the use of combustible cigarette. Therefore, a complete ban of e-cigarettes may not be appropriate. Instead, authorities could take a more tactful approach by maintaining the current status of e-cigarette smoking while regulating nicotine content and other chemicals in such products given evidence of health risks of e-cigarettes.

## Figures and Tables

**Table 1:** Factor loading and convergent validity

Constructs	Indicators		Outer loading	Cronbach’s alpha	Composite reliability	AVE
E-cigarette smoking behaviour (13)	EB1	I drop everything to go out and buy electronic cigarettes or e-juice	0.709	0.913	0.909	0.591
	EB2	I vape more before going into a situation where vaping is not allowed	0.752			
	EB3	When I haven’t been able to vape for a few hours, the craving gets intolerance	0.784			
	EB4	When I am craving an electronic cigarette, it feels like I’m in the grip of some unknown force that I cannot control	0.784			
	EB5	I crave vaping at certain times of day	0.763			
	EB6	My urges to vape keep getting stronger if I don’t vape	0.766			
	EB7	After not vaping for a while, I need to vape in order to avoid feeling any discomfort	0.766			
	EB8	It is hard to ignore urges to vape	0.780			
	EB9	When I go too long without vaping, I get strong urges that are hard to get rid of	0.813			
Perceived health benefit (14)	PH1	Electronic cigarette will improve breathing	0.738	0.733	0.832	0.554
	PH2	Electronic cigarette improves general wellbeing	0.777			
	PH3	Electronic cigarette decreases coughing	0.729			
	PH4	Electronic cigarette less likely to cause cancer	0.731			
Quit desire (14)	QD1	Using electronic cigarettes helps me to cut down or reduce the number of tobacco cigarettes smoked	0.793	0.851	0.890	0.574
	QD2	Using electronic cigarettes helps me to taking control over my addiction to tobacco cigarettes	0.740			
	QD3	Using electronic cigarettes helps me to tobacco cigarette smoking cessation	0.756			
	QD4	Using electronic cigarettes helps me to curb the craving for smoking tobacco cigarettes	0.748			
	QD4	Using electronic cigarettes helps me to get to the point where I can quit tobacco cigarettes forever	0.735			
	QD5	Using electronic cigarettes helps me to manage my tobacco cigarette smoking quit plan	0.772			
Social acceptance (14)	SA1	Compare to tobacco cigarette, using electronic cigarette is more accepted by most of the people I know	0.868	0.734	0.850	0.654
	SA2	Compare to tobacco cigarette, using electronic cigarette is more accepted by my friends	0.754			
	SA3	Compare to tobacco cigarette, using electronic cigarette is more accepted by my co-workers/classmates if you are studying	0.8			
Social impact (14)	SI1	I use electronic cigarette to be part of movement or something bigger than me	0.742	0.851	0.889	0.572
	SI2	I use electronic cigarette to change my social interactions and status for the better	0.774			
	SI3	I use electronic cigarette to not to feel like an outcast anymore	0.753			
	SI4	I use electronic cigarette a good conversation starter	0.777			
	SI5	I use electronic cigarette to opens doors for business/friendship	0.752			
	SI6	I use electronic cigarette to gain social acceptance	0.741			
Usefulness of product (14)	UP1	Electronic cigarette is discreet in use (no lingering smell, able hide to use)	0.794	0.868	0.901	0.602
	UP2	Electronic cigarette practical in use (no lighter, no ashtray, one puff, and able to store the device)	0.729			
	UP3	Electronic cigarette can be used in most weather conditions (rainy, windy, etc.)	0.811			
	UP4	Electronic cigarette no ashes to worry about	0.767			
	UP5	Electronic cigarette to avoid ruining my clothes (e.g., burn holes or smell that won’t washout)	0.761			
	UP6	Electronic cigarette more portable/mobile than other tobacco product I use	0.789			

Notes: EB= E-cigarette smoking behaviour; PH = perceived health benefit; QD = quit desire; SA = social acceptance; SI = social impact; UP = usefulness of product; AVE = average variance extracted

**Table 2:** Discriminant validity (HTMT)

	Construct	1	2	3	4	5
1	Perceived health benefit					
2	Quit desire	0.765				
3	E-cigarette smoking behaviour	0.773	0.659			
4	Social acceptance	0.853	0.847	0.805		
5	Social impact	0.79	0.727	0.845	0.831	
6	Usefulness of product	0.743	0.888	0.655	0.874	0.824

**Table 3:** Structural model result

Hypothesis	Relationship	Beta and *t*-values	*Q* ^2^	*f* ^2^	Result
H1	PH → EB	0.194 (3.053)**	0.369	0.051	Supported
H2	QD → EB	0.089 (1.162)		0.007	Not supported
H3	SA → EB	0.233 (4.338)**		0.060	Supported
H4	SI → EB	0.495 (7.068)**		0.275	Supported
H5	UP → EB	−0.108 (1.298)		0.010	Not Supported

Notes: EB = e-cigarette smoking behaviour; PH = perceived health benefit; QD = quit desire; SA = social acceptance; SI = social impact; UP = usefulness of product; significant at *P* < 0.01**
